# Various forms of tobacco usage and its associated oral mucosal lesions

**DOI:** 10.4317/jced.52654

**Published:** 2016-04-01

**Authors:** Boddu Naveen-Kumar, Ramesh Tatapudi, Reddy Sudhakara-Reddy, Satish Alapati, Kotha Pavani, Kotu-Nagavenkata Sai-Praveen

**Affiliations:** 1 Senior Lecturer, Department of Oral Medicine and Radiology, Sibar Institute of Dental Sciences, Takkellapadu, Guntur district, Andhra Pradesh, India; 2 Professor, Department of Oral Medicine and Radiology, Vishnu dental college, Bhimavaram, Andhra Pradesh, India; 3 Professor and head of the department, Department of Oral Medicine and Radiology, Vishnu dental college, Bhimavaram, Andhra Pradesh, India; 4 Senior Lecturer, Department of Oral Medicine and Radiology, St.Joseph Dental College, Elluru, Andhra Pradesh, India; 5 MDS, Department of Oral Medicine and Radiology, Bhimavaram, Andhra Pradesh, India; 6 Post graduate student, Department of Oral Medicine and Radiology, Vishnu dental college, Bhimavaram, Andhra Pradesh, India

## Abstract

**Background:**

To study the various forms of tobacco usage and its associated oral mucosal lesions among the patients attending Vishnu Dental College Bhimavaram.

**Material and Methods:**

An observational cross-sectional study was conducted in a total of 450 patients who were divided into three groups based upon type of tobacco use, as Group-1 Reverse smoking, Group-2 Conventional smoking, Group-3 Smokeless tobacco group and each group consists of 150 subjects.

**Results:**

Reverse smoking was observed to be more prevalent among old females with smoker’s palate and carcinomatous lesions being the most common. Conventional smoking was observed more in male patients with maximum occurrence of leukoplakia and tobacco associated melanosis. Smokeless tobacco habit was predominantly seen in younger males. Habit specific lesions like tobacco pouch keratosis, Oral Submucous Fibrosis (OSMF), Quid induced lichenoid reaction were noticed in smokeless tobacco habit group except for erythroplakia which was noticed only in conventional smoking group and it was not significant statistically.

**Conclusions:**

In the present study it was found that the usage of reverse smoking habit was most commonly seen in females and this habit is practiced in and surrounding areas of Bhimavaram with more occurrence of carcinoma compared to conventional smoking and smokeless tobacco.

** Key words:**Tobacco, reverse smoking, conventional smoking, smokeless tobacco, carcinoma.

## Introduction

Oral cancer is one of the most common cancers in India today and also stands among the ten most common cancers in the world. Tobacco, Alcohol and Betel usage are the main risk factors for Oral cancer development (1).

Tobacco is one of the most important cause of both addiction and development of Oral cancer. Christopher Columbus first discovered tobacco and was widely used in Europe at that time. Later Spanish and Portuguese sailors carried this tobacco to other parts of world. Initially tobacco is known as a plant with medicinal values and was used in various forms like tobacco ointments, pastes, mouth rinses etc., to treat various maladies (2).

Later it came into light that tobaccos possess various harmful substances. Thousands of chemical compounds are noticed in both smoked as well as un-burnt form of tobacco. They act not only as irritants and toxins, but also are deadly carcinogens. Nicotine which is an alkaloid is mainly responsible for addiction, whereas tobacco-specific nitrosamines, polycyclic aromatic hydrocarbons, and many others are most potent carcinogens (3).

India is next to China in both tobacco production and consumption in the world. In India tobacco is mainly used either in smoking form like cigarette, beedi, hookah, and other pipes like chillum, chutta, dhumti, cherrot and cigar or in smokeless form like chewing plain tobacco, khaini, zarda, kiwam, bajjar/tapkheer (dry snuff), masheri/mishri, and gutka. Products containing tobacco and areca nut are also used in some parts of the country. Among all the most commonly used one is chutta with cigarette next to it (4).

In West Godavari district, Andhra Pradesh, chutta or cigarette smoking is commonly seen among smoking form of tobacco. Cigarette smoking is noticed in upper middle class and high socioeconomic people, where as conventional chutta smoking, reverse chutta smoking is seen in low socioeconomic people. Reverse smoking is commonly seen in fishermen group and in agricultural labor.

Reverse smoking or Reverse chutta smoking is a form of usage of tobacco in which the lit end of the chutta is placed inside the mouth. It is most commonly seen in elder women among low socio-economic group in coastal region which was previously re-ported in Srikakulam district of Andhra Pradesh where in the prevalence of oral mucosal lesions is high (4).

The effects of tobacco on the oral mucosa ranges from initial mucosal changes to full blown oral cancer and these changes are intern dependent on various forms of tobacco usage. So with this background a study was conducted to notice the various mucosal lesions with different forms of tobacco habit usage among patients attending Vishnu Dental College, Bhimavaram.

## Material and Methods

An observational cross-sectional study was conducted after obtaining approval from institutional ethical committee (Vishnu Dental College, Bhimavaram). A total of 450 patients of various age groups with habit of tobacco usage (minimum of 6 months) attended to outpatient department of Vishnu dental college were included in the study. All the 450 patients were divided into three groups based upon their tobacco usage with 150 patients in each group.

Group 1: Reverse smokers

Group 2: Conventional smokers

Group 3: Smokeless tobacco

All the patients were explained about the details of the study and informed consent was obtained, those who were not interested to participate in the study, and those with Oral lesions not associated with tobacco were excluded from the study.

All the patients were examined by one oral medicine and radiology specialist and the details of them were recorded in a specially designed proforma which include demographic data, and personal history regarding various tobacco related habits. Clinical examinations were performed in a setting that had a fully reclining dental chair, direct and indirect light using diagnostic instruments.

Tobacco associated oral lesions like Leukoplakia, Erythroplakia, Smoker’s palate were diagnosed according to World Health Organization (WHO) criteria 1980 (5), Quid-induced lichenoid reaction according to Avon *et al.* (6), Oral Submucous Fibrosis (OSMF) according Kerr *et al.* (7), Tobacco associated melanosis according to Gualerzi *et al.* (8), and Carcinoma as described by Neville and Day (9).

The collected data was entered in a spread sheet (Excel 2007, Microsoft Office) and Minitab Version 16 SPSS software was used for statistical analysis. Descriptive statistical analysis was used along with Chi-square/ Fisher Exact test to find the significance of study parameters on categorical scale between the groups.

## Results

**Figure 1:**
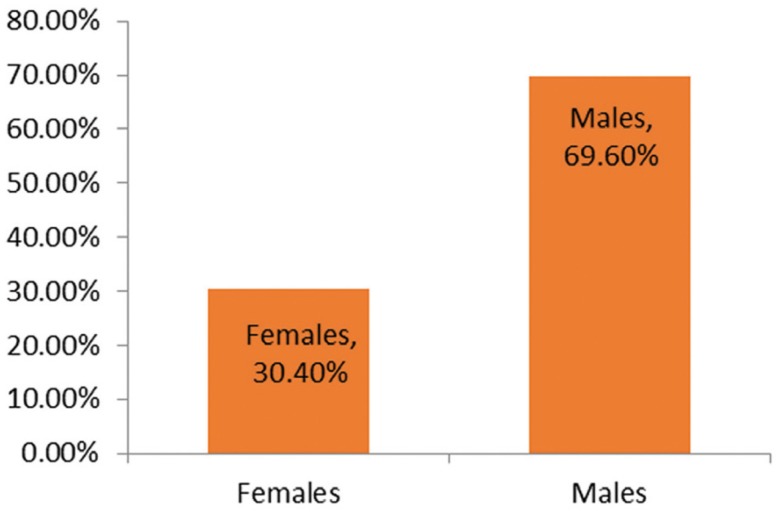
Bar diagram showing distribution of gender in total sample.

**Figure 2:**
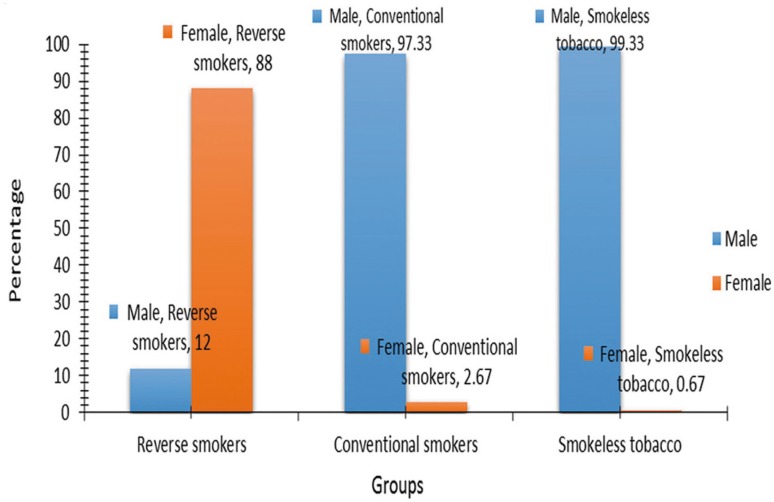
Bar diagram showing gender distribution among the tobacco habit groups.

Among the 450 patients enrolled in the study group 313 were males and 137 were females (Fig. 1) with a mean age of 52 years. Out of 137 females 132 were Reverse smokers, 4 were conventional smokers and one used smokeless form of tobacco. Out of 313 male subjects 143 were conventional smokers, 149 were using smokeless tobacco and 18 were reverse smokers (Fig. 2).

Most of the reported tobacco users were farmers accounting for about 16.8% of the total study population, followed by daily wagers and agriculture labor. Among the entire female sample most of them were household women followed by daily wagers and agriculture labors. The details regarding the occupation of study sample were shown in  Table 1.

**Table 1:**
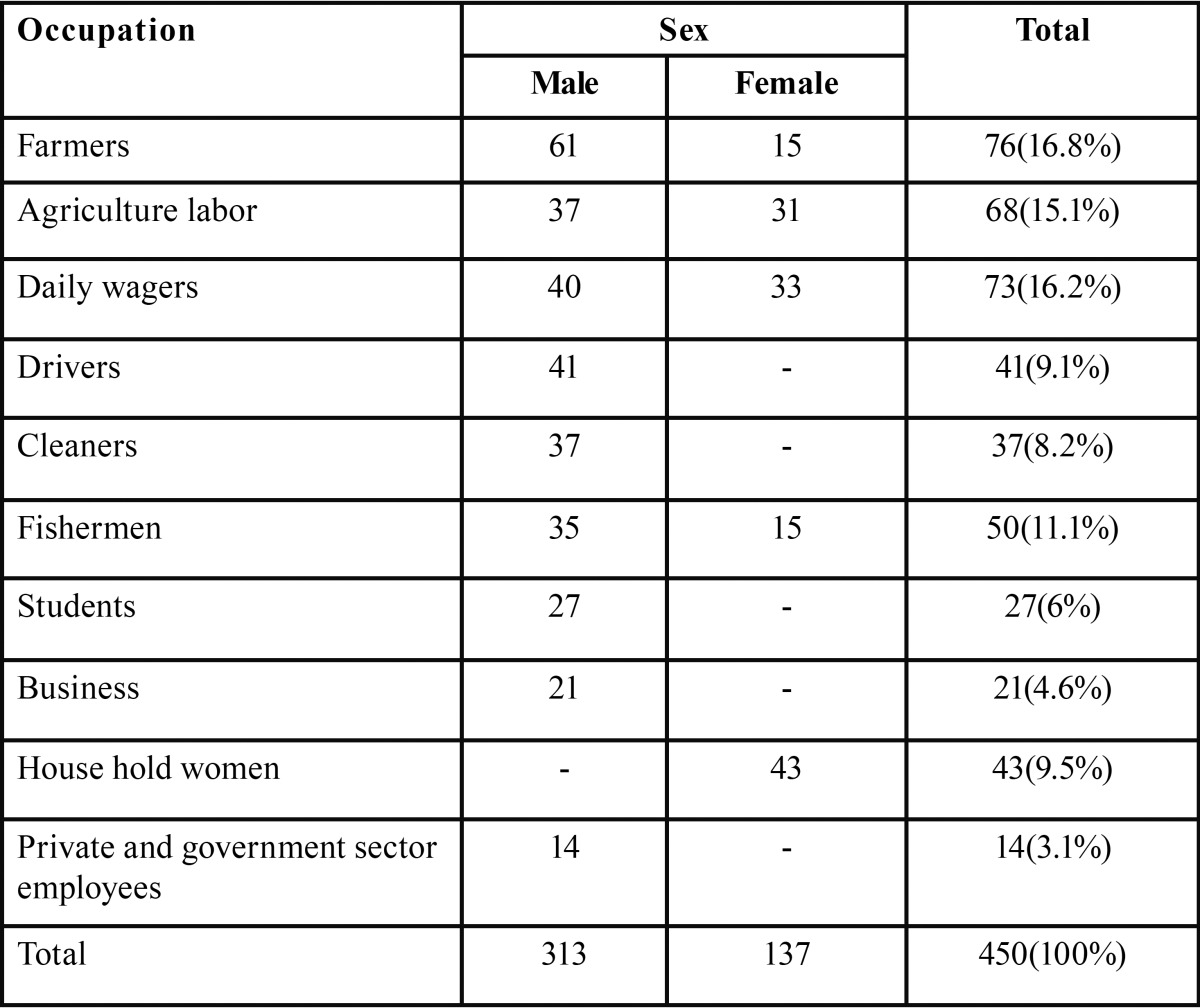
Occupational distribution among the study sample.

**Figure 3:**
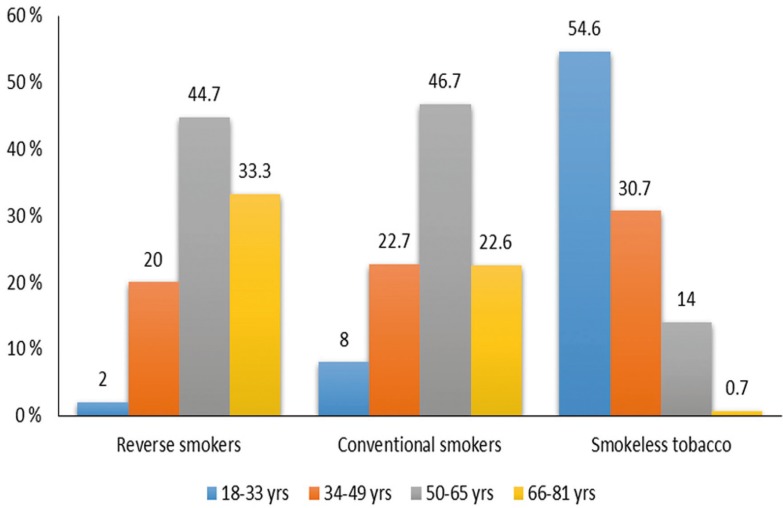
Bar diagrams showing distribution of Age groups among the tobacco habit groups.

Age distribution among the study groups were further divided into 4 sub-groups with 15 years of age range in each. Group-A included 97 patients in between the age group of 18-33 years, Group-B included 105 patients with age between 34-49 years, Group-C included 159 patients with age between 50-69 years, Group-D included 85 patients between 66-80 years (Fig. 3).

The frequencies of tobacco usage per day were again categorized into 4 sub-groups with subjects taking 1-5/day in Group-a, 6-10/day in Group-b, 11-15/day in Group-c, and 16-20/day in Group-d. The distribution of patients in various forms of tobacco usage based upon their frequency of usage was shown in the  Table 2. There was a significant statistical difference in chi-square test between tobacco habit groups and frequency of habit groups with Chi-Square value of 78.020, *p*-Value of 0.000.

**Table 2:**
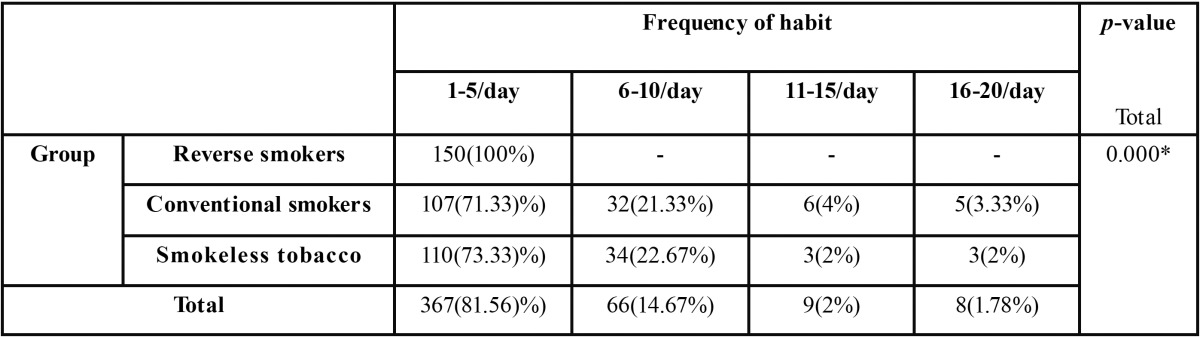
Analysis of the groups with tobacco habit and frequency of habit.

Based on the duration of habit patients were again divided into six sub-groups, with tobacco usage period of 1-10 years as Group-I, 11-20 years as Group-II, 21-30 years as Group-III, 31-40 years as Group-IV, 41-50 years as Group-V and 50-60 years as Group-VI. The distribution of patients in various forms of tobacco usage based upon their duration of tobacco usage was shown in the  Table 3. There was a significant statistical difference between tobacco habit groups and duration of habit groups with Chi-Square value of 53.263 and *p*-Value of 0.000.

**Table 3:**
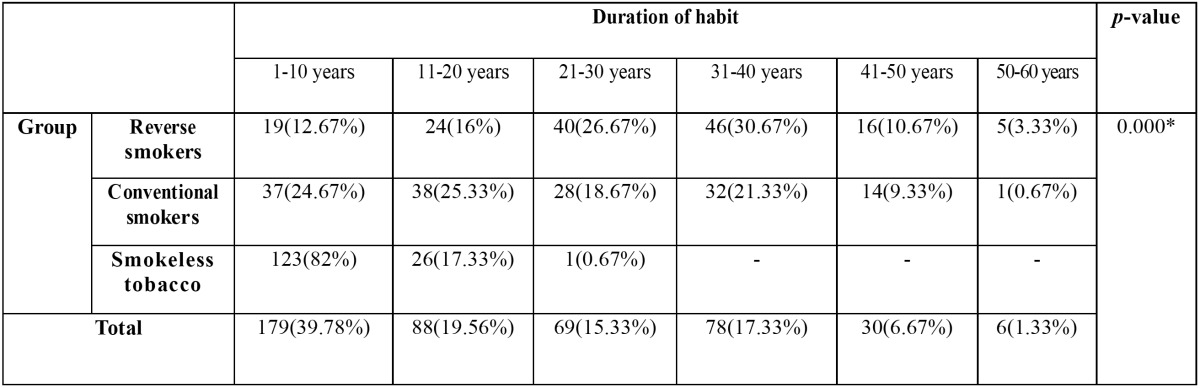
Analysis of the groups with tobacco habit and duration of habit.

There were a total of 684 oral lesions out of which 54 were leukoplakia, 4 erythroplakia, 23 tobacco pouch keratosis, 3 quid induced lichenoid reactions, 233 smokers palate lesions, 57 OSMF, 282 tobacco associated melanosis, and 28 were carcinoma cases. Of all the reported oral lesions most of them were in conventional smoking group 278(40.64%) followed by reverse smokers and smokeless tobacco habit group 275(40.2%) and 131(19.15%) respectively  Table 4.

**Table 4:**
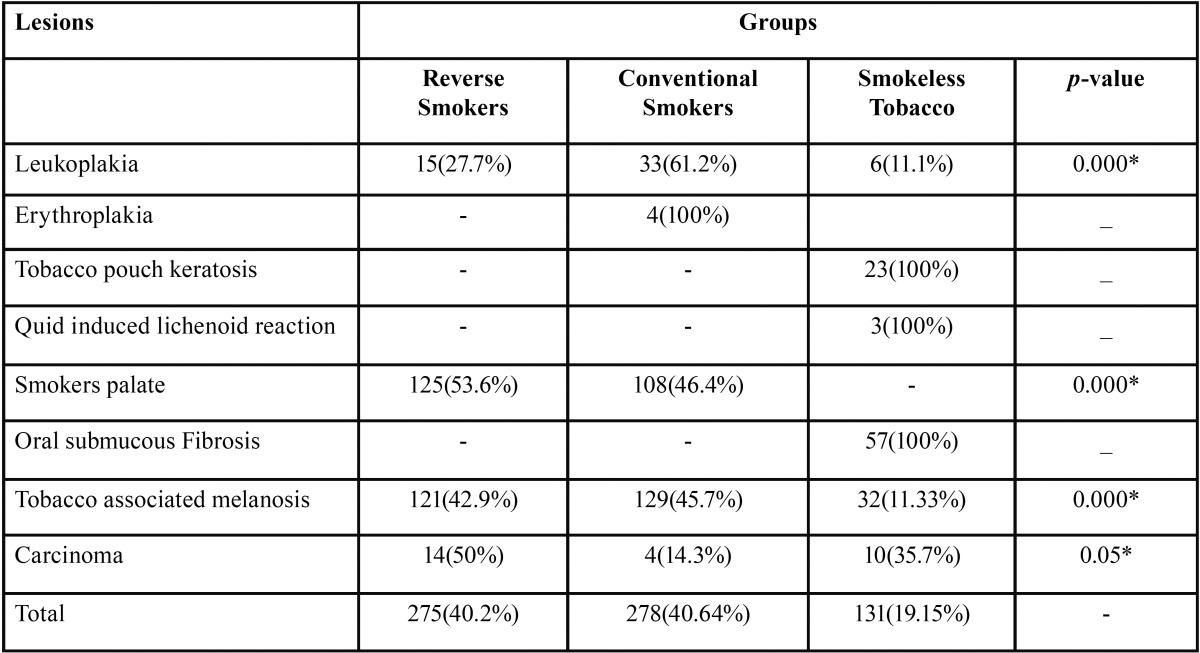
Distribution of the groups with tobacco habit and lesions.

Leukoplakia were observed more in conventional smokers group 33(61.2%) followed by reverse smoking group 15 (27.7%) and smokeless tobacco 6(11.1%) with significant *p*-value of 0.000. Erythroplakia were noticed only in conventional smoker group 4(100%).

Tobacco pouch keratosis, Quid induced lichenoid reaction, OSMF were identified only in smokeless tobacco group 23(100%), 3(100%), 57(100%) respectively. Smoker’s palate was noticed only in reverse smoking group and conventional smoking group 125(53.6%), 108(46.4%) respectively, but it was not found in smokeless tobacco group with significant *p*-value of 0.000.

Tobacco associated melanosis were observed more in conventional smokers 129(45.7%) followed by reverse smokers and smokeless tobacco group 121(42.7%) and 32(11.33%) respectively with significant *p*-value of 0.000. Carcinomas were noticed more in reverse smoking group 14(50%) followed by smokeless tobacco group and conventional smoking group 10(35.7%) and 4(14.3%) respectively with significant *p*-value of 0.05.

## Discussion

Occurrence of tobacco associated oral mucosal lesions varies with the form of tobacco usage. Hence there was a need for a study to know the diversity in tobacco usage and their associated oral mucosal lesions. With this background a hospital based observational cross-sectional study was conducted to identify various oral mucosal lesions among tobacco related habits in and surrounding areas of Bhimavaram.

Most of the tobacco users in the present study were farmers, agriculture labor, daily wagers, drivers, cleaners and fishermen. Most of these occupations require high physical energy and are also associated with high level of physical stress which in combination with peer pleasure can lead to initiation of deleterious oral habits like use of tobacco.

Out of 137 female patients in the study most of them (88%) were reverse smokers and this finding was in accordance with studies conducted by Mehta *et al.* (4), and Ramulu *et al.* (10). The female predominance in reverse smoking observed in this study can be attributed to reasons like intention to hide it from their husbands, to take precautions against splashes of water may put off chutta when fishermen and women work in water, to prevent hot ashes falling on children and clothes at the time of nursing, and a few taboos considering it as a treatment for toothache as it causes soothing sensation.

Male patients were predominant in both conventional type and smokeless type which was in accordance with studies conducted by Saraswathi *et al.* (11), and Ray *et al.* (12), whereas Female predominance was seen in studies conducted by Lima *et al.* (13).

Most of the patients in both conventional and reverse smoking group were between 50-65 years of age, whereas majority of patients in smokeleless tobacco group were in the age range of 18-33 years. In the present study frequency of tobacco usage in each habit group showed a mean of 1-5 units per day.

In the present study we had noticed, a total number of 684 oral lesions associated with tobacco usage out of which 282 were tobacco induced melanosis with 121 cases in reverse smoking group, 129 in conventional group and 32 cases in smokeless group. Smoking induces increased melanin pigmentation in the oral mucosa which may be due to the effect of nicotine on melanocytes located along the basal cells, which results in basilar melanosis with varying amounts of melanin incontinence. Whereas pigmen-tation at the site of quid placement was absent with mild pigmentation noticed away from the site of quid placement in smokeless tobacco form. It can be hypothesized that the mechanical and chemical irritation from smokeless tobacco may have induced melanin pigmentation (14).

Smoker’s palate stands next to melanosis with a total number of 233 cases in which 125 cases were seen in reverse smokers and 108 in conventional smokers with mere absence in smokeless tobacco usage. Smoker’s palate lesions were noticed more with habit of smoking. According to Axell *et al.* (15) smoker’s palate is probably related more to the high temperature rather than the chemical composition of the smoke, although there is a synergistic effect of the two. So it is not observed in smokeless tobacco habit group.

A total of 54 leukoplakia cases were reported out of which 33 were from conventional smoking group, 15 in reverse smoking group and 6 from smokeless tobacco group. Erythroplakia had been exclusively noticed only in conventional group (4 cases). Whereas lesions like tobacco pouch keratosis, quid induced lichenoid reaction and OSMF are exclusively seen in smokeless tobacco group. According to Axell *et al.* (15) tobacco pouch keratosis may occur due to chronically stretched tissues in the area of tobacco placement and the lesion is confined to areas in direct contact with spit tobacco.

Out of 28 carcinoma cases 50% were from reverse smoking group followed by smokeless tobacco group and conventional smoking group with an incidence rate of 35.7% and 14.3% respectively.

In reverse smokers the smoke emitted from chutta is in direct contact to oral mucosa. This emitted smoke contains high concentration of alkaline pH, which facilitates absorption of substances like nicotine alkaloid, reducing sugars, and nitrogen. Apart from this it also causes increase in internal temperature of about 760°C, and intraoral air upto 120°C. These temperature variations also act as co-carcinogens. These changes are responsible for increased incidence of carcinoma in the oral cavity when compared with conventional smokers (14).

The common lesions that were in conventional smoking group are leukoplakia, and erythroplakia, whereas in smokeless tobacco habit group lesions such as leukoplakia, and exclusively tobacco pouch keratosis, Quid-induced Lichenoid reaction and OSMF were noticed.

It is established in this study that reverse smoking habit is common in older females and are more prone for malignancy compared to smokeless tobacco and conventional smoking habit. Efforts should be taken to educate individuals and masses regarding the risk factors of tobacco usage.
